# A connectionist model of context-based memory reconsolidation in the hippocampus: the role of sleep

**DOI:** 10.1186/1471-2202-15-S1-P163

**Published:** 2014-07-21

**Authors:** Justin Lines, Kelsey Nation, Jean-Marc Fellous

**Affiliations:** 1 Department of Psychology, University of Arizona, Tucson, Arizona 85721, USA; 2 Neuroscience Graduate Interdisciplinary Program, University of Arizona, Tucson, Arizona 85719, USA; 3 Program in Applied Mathematics, University of Arizona, Tucson, Arizona 85721, USA

## 

Context-based memory reconsolidation has been studied in human and animal models [1]. In these paradigms, subjects learn two lists of items on two different days and are asked to recall the first list on day 3. Subjects who learn the two lists in the same spatial context make significantly more errors on day 3 than subjects who learn the lists in different contexts. This result suggests that contextual information may be linked to item information during memory formation or consolidation, and that this link is responsible for intrusions of items from the second list into the first list during recall when the lists were learned in identical contexts. The neural mechanisms underlying this process are unknown, but experimental studies have suggested that the hippocampus may be critical.

**Figure 1:**
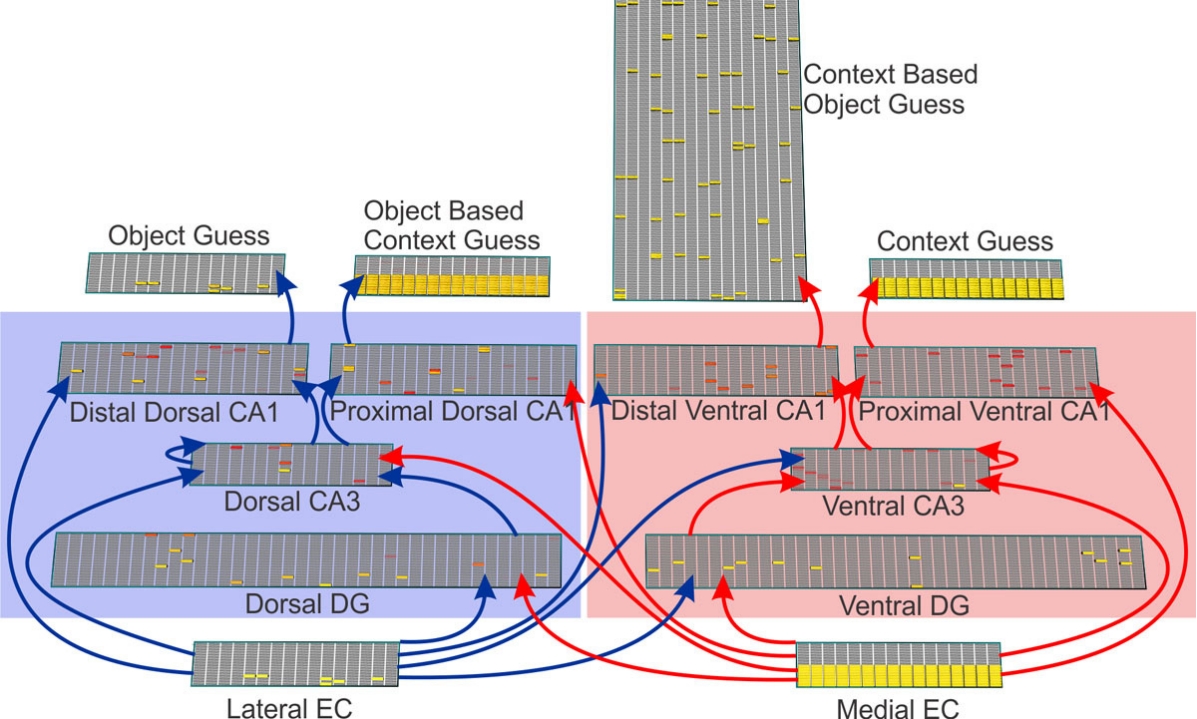
Model architecture. Separate and interacting ‘object’ and ‘context’ streams shown in blue and red respectively, with explicit output ‘guesses’.

Experimental work has shown that the dorsal and ventral portions of the hippocampus may implement qualitatively different functions in memory and spatial navigation [2], and that the proximal and distal portions of CA1 may carry information related to self-motion and sensory perception respectively [3]. We hypothesize that the dorso-ventral and proximal-distal anatomical differentiations of this structure may explain some of the experimental data on memory reconsolidation. To test this hypothesis, we built a connectionist model of the hippocampus (figure 1). The model is implemented using EMERGENT [4]. In this model, the dorsal stream carries predominantly item information, while the ventral stream carries spatial contextual information. In both streams, the distal CA1 encodes items using inputs from the lateral entorhinal cortex, while the proximal CA1 encodes spatial context using medial entorhinal cortical inputs. We train and test the model as in the experiments.

We found that object representation overlap as well as additional, extraneous learning can explain how context affects recall performance and produce intrusions as observed experimentally. We then selectively lesion the network to investigate which component of the hippocampus affects context based memory recall. In addition, we use the model to understand memory reactivation during sleep. Sleep is simulated by presenting small amounts of noise in the input layers. We found that this noise partially re-activated the memory representations of objects that were previously learned. These partial memories were then set as inputs and were re-processed by the network. This in turn made these memories resilient to interference from new items learned at a later time as was shown experimentally [5]. The model will allow for an investigation of why certain items have preferential memory reactivation during sleep. Furthermore, the model may be used to explain recent experimental data showing that presenting specific external stimuli during sleep may influence the memory consolidation process [6].
